# The interleukin-11 receptor variant p.W307R results in craniosynostosis in humans

**DOI:** 10.1038/s41598-023-39466-y

**Published:** 2023-08-18

**Authors:** Ilyas Ahmad, Juliane Lokau, Birte Kespohl, Naveed Altaf Malik, Shahid Mahmood Baig, Roland Hartig, Daniel Behme, Roland Schwab, Janine Altmüller, Muhammad Jameel, Sören Mucha, Holger Thiele, Muhammad Tariq, Peter Nürnberg, Jeanette Erdmann, Christoph Garbers

**Affiliations:** 1 Institute for Cardiogenetics, and University Heart Center, University of Lübeck, Building 67, BMF, Ratzeburger Allee 160, 23562 Lübeck, Germany; 2 DZHK (German Research Center for Cardiovascular Research), Partner Site Hamburg/Lübeck/Kiel, 23562 Lübeck, Germany; 3 Cologne Center for Genomics (CCG) and Center for Molecular Medicine Cologne (CMMC), Faculty of Medicine and University Hospital Cologne, University of Cologne, 50931 Cologne, Germany; 4 Department of Pathology, Medical Faculty, Otto-Von-Guericke-University, 39120 Magdeburg, Germany; 5 National Institute for Biotechnology and Genetic Engineering College, Pakistan Institute of Engineering and Applied Sciences (NIBGE-C, PIEAS), Faisalabad, 38000 Pakistan; 6 Department of Biological and Biomedical Sciences, Aga Khan University, Karachi, 74800 Pakistan; 7 Institute for Molecular and Clinical Immunology and Service Unit Multiparametric Bioimaging and Cytometry, Medical Faculty, Otto-Von-Guericke-University, 39120 Magdeburg, Germany; 8 University Clinic for Neuroradiology, Medical Faculty, Otto-Von-Guericke-University, 39120 Magdeburg, Germany; 9 Core Facility Genomics, Berlin Institute of Health at Charité - Universitätsmedizin Berlin, Berlin, Germany; 10 Max Delbrück Center for Molecular Medicine in the Helmholtz Association (MDC), Berlin, Germany; 11 Centre for Regenerative Medicine and Stem Cell Research, Aga Khan University, Karachi, 74800 Pakistan; 12 Institute of Epidemiology, Kiel University, 24105 Kiel, Germany; 13 Health Campus Immunology, Infectiology and Inflammation (GC:I3), Otto-Von-Guericke-University, 39120 Magdeburg, Germany; 14 Center for Health and Medical Prevention (ChaMP), Otto-Von-Guericke-University, 39120 Magdeburg, Germany; 15 Institute of Clinical Biochemistry, Hannover Medical School, Hannover, Germany

**Keywords:** Biochemistry, Genetics

## Abstract

Craniosynostosis is characterized by the premature fusion and ossification of one or more of the sutures of the calvaria, often resulting in abnormal features of the face and the skull. In cases in which growth of the brain supersedes available space within the skull, developmental delay or cognitive impairment can occur. A complex interplay of different cell types and multiple signaling pathways are required for correct craniofacial development. In this study, we report on two siblings with craniosynostosis and a homozygous missense pathogenic variant within the *IL11RA* gene (c.919 T > C; p.W307R). The patients present with craniosynostosis, exophthalmos, delayed tooth eruption, mild platybasia, and a basilar invagination. The p.W307R variant is located within the arginine-tryptophan-zipper within the D3 domain of the IL-11R, a structural element known to be important for the stability of the cytokine receptor. Expression of IL-11R-W307R in cells shows impaired maturation of the IL-11R, no transport to the cell surface and intracellular retention. Accordingly, cells stably expressing IL-11R-W307R do not respond when stimulated with IL-11, arguing for a loss-of-function mutation. In summary, the IL-11R-W307R variant, reported here for the first time to our knowledge, is most likely the causative variant underlying craniosynostosis in these patients.

## Introduction

The formation of the skull is a highly regulated and unique developmental process^1^. Via a process that is called intramembranous ossification, the calvarial bones form from multipotent mesenchymal cells that proliferate and subsequently differentiate into osteoblasts^2^. Flexible structures, called sutures, form between and line the developing calvaria and thereby allow development and expansion of the skull alongside the growth of the underlying brain^2,3^. A tight balance between proliferation, differentiation and apoptosis ensures the continuous flexibility of the sutures, which is maintained until the skull formation is complete, after which the sutures close^4^. Importantly, while the metopic suture fuses within the first year of life, the remaining sutures are patent until early adulthood.

Distortion of this process usually leads to the premature closure of one or multiple sutures and causes a condition that is called craniosynostosis. Craniosynostosis is the second most common craniofacial abnormality. Initially described in newborn children at a rate of 1:2,500^2,5^, newer epidemiological data found an increased incidence of 1:1800^6^ or even 1:1400^7^. Until today, pathogenic variants in more than 90 genes have been described that cause craniosynostosis^4^. The dominant pathway appears to be the fibroblast growth factor (FGF) signaling cascade. Variants in the genes *FGFR1*, *FGFR2* and *FGFR3*, which encode three of the four FGF receptors, are reported to cause craniosynostosis^8^. However, also mutations in other genes, including *TWIST1*, *MSX2* and *RAB23* have been reported in patients with craniosynostosis^9,10^. Intriguingly, which suture in particular closes prematurely appears to be influenced by the causatively mutated gene. However, our understanding of how and why these different pathways affect the different sutures and which molecular interplay between them exists is far from complete^2^.

Beginning in 2011, several reports have been published linking pathogenic variants within the *IL11RA* gene to craniosynostosis ([MIM: 614188]^11–17^, for an overview see^18^). Variants in *IL11RA* have been described in patients with heterogenous clinical phenotypic presentation (syndromic and non-syndromic), with inter- and intrafamilial variations revealing a complex genotype–phenotype relationship in patients. Non-syndromic patients do not shown additional dysmorphisms of the face, trunk, or extremities, and non-syndromic craniosynostoses usually involve only a single suture^19^. *IL11RA*-linked disease phenotypes include synostosis of the calvarial bones, maxillary and midface hypoplasia, variable degree of exophthalmos, conductive hearing loss, delayed and ectopic tooth eruption, formation of supernumerary teeth, and digital anomalies^11,12^. This gene encodes the interleukin-11 (IL-11) receptor (IL-11R), which is a type-I transmembrane protein. Its extracellular part consists of an Ig-like domain (termed D1) and two fibronectin-type-III-like domains (termed D2 and D3), which are connected via a stalk region to a transmembrane helix and a short intracellular region^20^. The D2 and D3 domains are crucial for binding of the ligand IL-11, and the D3 domain contains the typical WSXWS-motif found in many different cytokine receptors^21,22^. The stalk region contains different cleavage sites for proteases^23,24^ and acts as a spacer to ensure the correct positioning of the three extracellular domains within the signaling complex^25^. IL-11 is a member of the IL-6 family of cytokines^26,27^ and plays important roles in several diseases, including breast cancer^28,29^, gastrointestinal tumorigenesis^30^, different fibrosis endotypes^31,32^ but also classical inflammatory diseases like arthritis^33,34^. To activate its target cells, IL-11 initially binds to the non-signaling IL-11R, and the resulting IL-11/IL-11R complex then induces the formation of a homodimer of the signal-transducing receptor gp130, which is shared with other members of the cytokine family^27^. Besides this classical signaling pathway of IL-11, trans-signaling via soluble forms of the IL-11R, which are generated by proteolytic cleavage of the membrane-bound IL-11R, has been reported^24,35,36^. Recently, the pathogenic variant p.R281Q within gp130 with a selective loss of IL-11 signaling has been reported in a craniosynostosis patient^37^. Using transfected cell lines and primary cells derived from the patient, it was shown that gp130-R281Q is unable to transmit signals when activated by IL-11, whereas signaling by other family members of the IL-6 family were unaffected, highlighting the slightly different gp130 signaling complexes induced by different cytokines^38^. IL-11 signaling is required for normal bone turnover and normal trabecular bone mass^39^ and is in general considered an essential factor for bone homeostasis^18,40^.

For the majority of craniosynostosis-associated IL-11R variants, it is currently unknown how they affect the receptor, what the underlying molecular mechanisms are and how this translates into the craniosynostosis phenotype. We have previously shown that the p.R296W variant, which is one of the variants described in the initial publication linking IL-11R variants to craniosynostosis^11^, results in the absence of the IL-11R from the cell surface^41^. Although IL-11R-R296W is expressed by cells at comparable levels as the IL-11R wildtype (IL-11R-WT), the mutant protein is retained within the endoplasmic reticulum, most probably due to a folding defect of the D3 domain, does not mature properly and thus results in the absence of any IL-11 signaling. We could further show that R296 is part of the arginine-tryptophan-zipper, which represents an important structural motif found in several cytokine receptors and is known to be crucial for receptor folding and thus receptor function^21,41^. Importantly, not all recessive variants in the IL-11R result in a loss-of-function effect in humans, as we have recently shown for different benign variants found in the gnomAD database^42^.

In the present study, we describe two siblings with a novel pathogenic variant within the IL-11R that suffer from craniosynostosis. Both patients are homozygous for the missense variant p.W307R located within the D3 domain. We show that IL-11R-W307R is expressed by cells, does not mature properly, is not transported to the cell surface and results in a complete loss of IL-11 signaling. Mechanistically, W307 is part of the arginine-tryptophan-zipper located in domain D3, and insertion of a arginine residue at position 307 disrupts this important structural feature, probably resulting in missfolding and, therefore, intracellular retention of the IL-11R.

## Materials and methods

### Subjects and clinical evaluation

The family of the two patients originates from a rural area in the southern Punjab province of Pakistan and belongs to the Baloch ethnic group. Clinical evaluation included physical examination, detailed family and personal history, neuroimaging and an orthopantomography (OPG) radiograph of the upper and lower jaws. This study complies with the Declaration of Helsinki and was approved by the Research Ethics Committee of the National Institute for Biotechnology and Genetic Engineering (NIBGE), Faisalabad, Pakistan. Written informed consent was obtained from the patients and their parents. The parents provided informed consent for the publication of photographs of the individuals. Genomic DNA from the affected individuals and family members was extracted from whole blood by standard methods.

### Exome capture and sequencing

Whole exome capture and sequencing were performed on individual (V:4) at the Cologne Center for Genomics (CCG), Cologne, Germany. All exons and adjacent intronic boundaries were captured by the SeqCap EZ Human Exome Library v2.0 kit from NimbleGen (Roche NimbleGen Inc., Madison, WI 53,719, USA) and subsequently run on an Illumina HiSeq™ 2000 sequencer. Briefly, one microgram of genomic DNA was fragmented by ultrasonication (Covaris, Woburn, MA, USA) according to the manufacturer's protocol. The fragments were end-repaired and adapter-ligated. After size selection, libraries were subjected to the enrichment process. For this sample, approximately 10 Gb of the sequence data was generated by loading it separately onto a flow cell lane and generating 2 × 100 bp paired-end reads. After sequencing, the data were analyzed as previously described^43,44^. Primary data were filtered for signal purity using Illumina Realtime Analysis (RTA) software v1.8. Reads were then mapped to the human genome reference build GRCh38/hg20 using the Burrows-Wheeler Aligner (BWA)  algorithm. GATK v1.64 was used to mark PCR duplicates and the output was converted to BAM format. Local re-alignment of poorly mapped regions and around insertion-deletion sites was executed with Genome Analysis Toolkit Indel Realigner v3.6. Single nucleotide polymorphisms (SNPs) and short insertions/deletions (INDELs) were called using the Platypus (v0.8.1), Haplotype Caller (v3.6), and SAMtools mpileup (v1.6) programs, followed by calibration using variant quality score calibration (VQSR) with GATK v3.6. The mean coverage in the exome was 66× , and 96.5% and 83.8% of the target bases were covered more than 10× and 30× , respectively.

Further annotation and filtering for high-quality rare variants (MAF < 0.1%) with a likely effect on protein sequence or splicing was performed using the “Varbank” exome and genome analysis pipeline v2.6 (https://varbank.ccg.uni-koeln.de/varbank2/) of the Cologne Center for Genomics (CCG, University of Cologne, Germany). The highest priority variants were validated by Sanger sequencing in all family members from whom blood samples were available. The chromatogram of each individual was visualized and meticulously scanned with DNASTAR Lasergene12 (DNASTAR Inc., Madison, WI, USA) to determine the genotype. Exon 9 of *IL11RA* was amplified with primers 5′-gcaaggggtgctctttgtag-3′ and 5′-acctgcctgtcttttggaac-3′.

### Cell lines

HEK293, Phoenix-Eco, and HeLa cells were cultured in DMEM high glucose medium (PAN-biotech, Aidenbach, Germany) containing 10% fetal bovine serum, penicillin (60 mg/l), and streptomycin (100 mg/l). Ba/F3-gp130 cells have been described previously^45^ and were cultured in DMEM high glucose medium (PAN-biotech, Aidenbach, Germany) containing 10% fetal bovine serum, penicillin (60 mg/l), and streptomycin (100 mg/l) and additionally supplemented with 10 ng/ml Hyper-IL-6^46^. All cells were kept at 37 °C and 5% CO_2_ in a humidified incubator.

### Antibodies and proteins

IL-11 and Hyper-IL-6, a fusion protein consisting of IL-6 linked to the soluble IL-6R, were produced in-house as described previously^46,47^. The primary antibodies for western blots α-Myc-tag (9B11), α-pSTAT3 (Tyr705, D3A7), α-STAT3 (124H6) and α-GAPDH (14C10) were from Cell Signaling Technology (Frankfurt, Germany). The IRDye-conjugated secondary antibodies IRDye800CW goat-α-rabbit, IRDye800CW donkey-α-mouse, IRDye800CW donkey-α-rabbit, IRDye680RD donkey-α-mouse, and IRDye680RD donkey-α-rabbit were from LICOR Biosciences (Lincoln, NE, USA). For flow cytometry, the α-Myc-tag (71D10, Cell Signaling Technology, Frankfurt, Germany) antibody and the AlexaFluor488-conjugated goat-α-rabbit (Thermo Fisher Scientific, Waltham, MA, USA) were used. For microscopy, the following antibodies were used: α-myc-tag (9B11) from Cell Signaling Technology (Frankfurt, Germany), and secondary AlexaFluor488-conjugated goat-α-mouse and AlexaFluor568-conjugated goat-α-rabbit antibodies (both from Thermo Fisher Scientific, Waltham, MA, USA).

### Expression plasmids

pcDNA3.1 and pMOWS(puro) expression plasmids containing the Myc-tagged IL-11R WT have been described previously^48^. The p.W307R mutation was introduced using splicing by overlapping extension PCR (SOE-PCR) and the mutated ORF was ligated into the expression plasmid using the NEBuilder HiFi DNA assembly kit (New England Biolabs, Ipswich, MA, USA). Sequences were confirmed by Sanger sequencing (Eurofins Genomics, Luxemburg, Luxemburg).

### Transfection and lysis of cells

HEK293 cells were transiently transfected with pcDNA3.1 expression plasmids encoding either the IL-11R WT or the IL-11R-W307R mutant, or an empty vector as mock control, using Turbofect (Thermo Fisher Scientific, Waltham, MA, USA) according to the manufacturer’s instructions. After 48 h, the cells were harvested and lysed (10 mM Tris–HCl pH 7.5, 150 mM NaCl, 50 mM EDTA, 1% Triton-X 100, and protease inhibitor cocktail (Sigma-Aldrich, St. Louis, USA)) prior to analysis by western blot.

### Stable transduction of Ba/F3-gp130 cells

The retroviral transduction of Ba/F3-gp130 cells has been described before^41^. In brief, pMOWS(puro) expression plasmids encoding either the IL-11R WT or the IL-11R-W307R mutant, or GFP as control, were transiently transfected into Phoenix-Eco cells. The next day, Ba/F3-gp130 cells were centrifuged with cell-free supernatant of the Phoenix-Eco cells and 8 µg/ml polybrene at 800×G for 2 h. Afterwards, the stably transfected cells were selected with 1.5 µg/ml puromycin.

### Stimulation of Ba/F3-gp130 derived cell lines

The Ba/F3-gp130 derived cell lines stably expressing either the IL-11R WT or IL-11R-W307R, or GFP as control, were washed three times with PBS and starved in serum-free medium for 2 h. Afterwards, cells were stimulated with 10 ng/ml IL-11 or Hyper-IL-6 for 15 min at 37 °C. The cells were centrifuged, boiled in Laemmli-buffer, and STAT3 phosphorylation was determined by western blot.

### Western blotting and densitometric analysis

Cell lysates were separated by SDS-PAGE and transferred onto nitrocellulose membranes. The membranes were blocked using 5% BSA in TBS for 1 h at room temperature. Then, membranes were incubated with the primary antibodies diluted 1:1000 in 1% BSA/TBST at 4 °C overnight. After washing three times with TBST, the membranes were treated with the matching IRDye-conjugated secondary antibodies diluted 1:10,000 in 1% BSA/TBST for 1 h at room temperature in the dark. After washing once with TBST and three times with TBS, fluorescence signals were detected using the ChemiDoc™ MP Imaging System (BioRad, Hercules, CA, USA). For quantification of STAT3 phosphorylation, the signal intensities of total and phosphorylated (p)STAT3 were analyzed using the ImageStudio Lite software (Li-COR Biosciences, Lincoln, USA) and the pSTAT3/STAT3 ratio from 3 independent experiments was calculated.

### Flow cytometry

For surface staining, Ba/F3-gp130 derived cell lines or transiently transfected HEK293 cells were washed with PBS and stained with α-Myc-tag antibody in 1% BSA/PBS for 1 h at 4 °C. Afterwards, cells were washed with 1%BSA/PBS and incubated with the AlexaFluor488-conjugated α-rabbit antibody for 1 h at 4 °C in the dark. For intracellular staining, the cells were washed with PBS and fixed in 4% PFA for 20 min at room temperature. Afterwards, the cells were again washed with PBS and permeabilized using 0.2% saponin in 1% BSA/PBS for 15 min at room temperature. After that, the cells were stained as described above but with the addition of 0.2% saponin to all buffers. Finally, all cells were washed twice in 1% BSA/PBS and analyzed using a flow cytometer (LSRFortessa™ cell analyzer, BD, Franklin Lakes, NJ, USA) and FlowJo Software (FlowJo, LLC). The geometric mean fluorescence intensity (gMFI) for each sample was normalized to the respective IL-11R WT sample.

### Cell viability assays

To analyze the viability of Ba/F3-gp130 derived cell lines in response to IL-11 or Hyper-IL-6, 5000 cells per well were seeded into 96-well plates and treated in triplicates with increasing amounts of the cytokines (0–100 ng/ml). After two days, the CellTiter Blue Viability Assay (Promega, Fitchburg, WI, USA) was added according to manufacturer’s instructions and the fluorescence intensity was measured on a CLARIOstar plate reader (BMG Labtech, Ortenberg, Germany) and normalized to the value measured at the starting point. Relative cell viability is presented as relative light units (RLU).

### Microscopy

The immunofluorescence staining was performed in principle as described previously^42^. In brief, 1 × 10^6^ HeLa cells were seeded on coverslips and transfected the following day as described above, after the medium was replaced by 5 ml DMEM. After 48 h cells were fixed with 4% paraformaldehyde/PBS containing 0.02% glutaraldehyde for 15 min at room temperature, washed three times with Hank’s Balanced Salt Solution (HBSS) and stained with WGA640R (Biotium, Fremont, CA, USA) (20 µg/ml in HBSS) for 10 min at room temperature. Cells were washed twice with HBSS and were either permeabilized with 0.12% Glycine/0.2% Saponin in PBS for 10 min or left untreated as unpermeabilized control. For further staining of the permeabilized cells, 0.2% Saponin was added to the blocking solution. Afterwards, cells were washed three times with PBS, blocked with 1% BSA/PBS for 10 min, washed again three times with PBS, before the cells were stained with anti-myc-tag (1:200 in 1% BSA/PBS) for 1 h at room temperature. Cells were washed three times with PBS, blocked with 1% BSA/PBS for 10 min and incubated with secondary antibody (1:300 in 1% BSA/PBS) for 1 h at room temperature. Afterwards, cells were washed three times with PBS and post fixed with 4% paraformaldehyde/PBS and 0.05% glutaraldehyde. After further washing with PBS and once with PBS pH 8.9, cells were mounted with Vectashield mounting medium containing DAPI (Vector Laboratories, Burlingame, CA, USA) and glycerol (1:1). Stained cells were visualized using an inverted Confocal Microscope System Leica SP8 (Leica Microsystems, Mannheim, Germany) containing a Plan Apo 63x/1.4 oil objective and controlled by LASX software (Leica). The detailed settings have been described previously^42^.

### Structural analysis

UCSF chimera was used to visualize the crystal structure of the IL-11R (6O4P) and perform the in silico point mutation^49,50^.

### Presentation of experimental data and statistical analyses

Western blots are presented as one example out of three independent experiments with similar outcome and, to analyze STAT3 phosphorylation, complemented by the quantification of the three independent experiments. For flow cytometry data, the normalized gMFI of three independent experiments are shown alongside one exemplary intensity histogram. Immunofluorescence data and viability assays are shown as one example out of three independent experiments with similar outcome. Statistical analysis was performed with GraphPad Prism 8 (GraphPad Software, San Diego, CA, USA) using one sample T test to analyzed differences between IL-11R-WT and IL-11R-W307R. Further details are given in the respective figure legends.

## Results

### Clinical description

**Figure 1:**
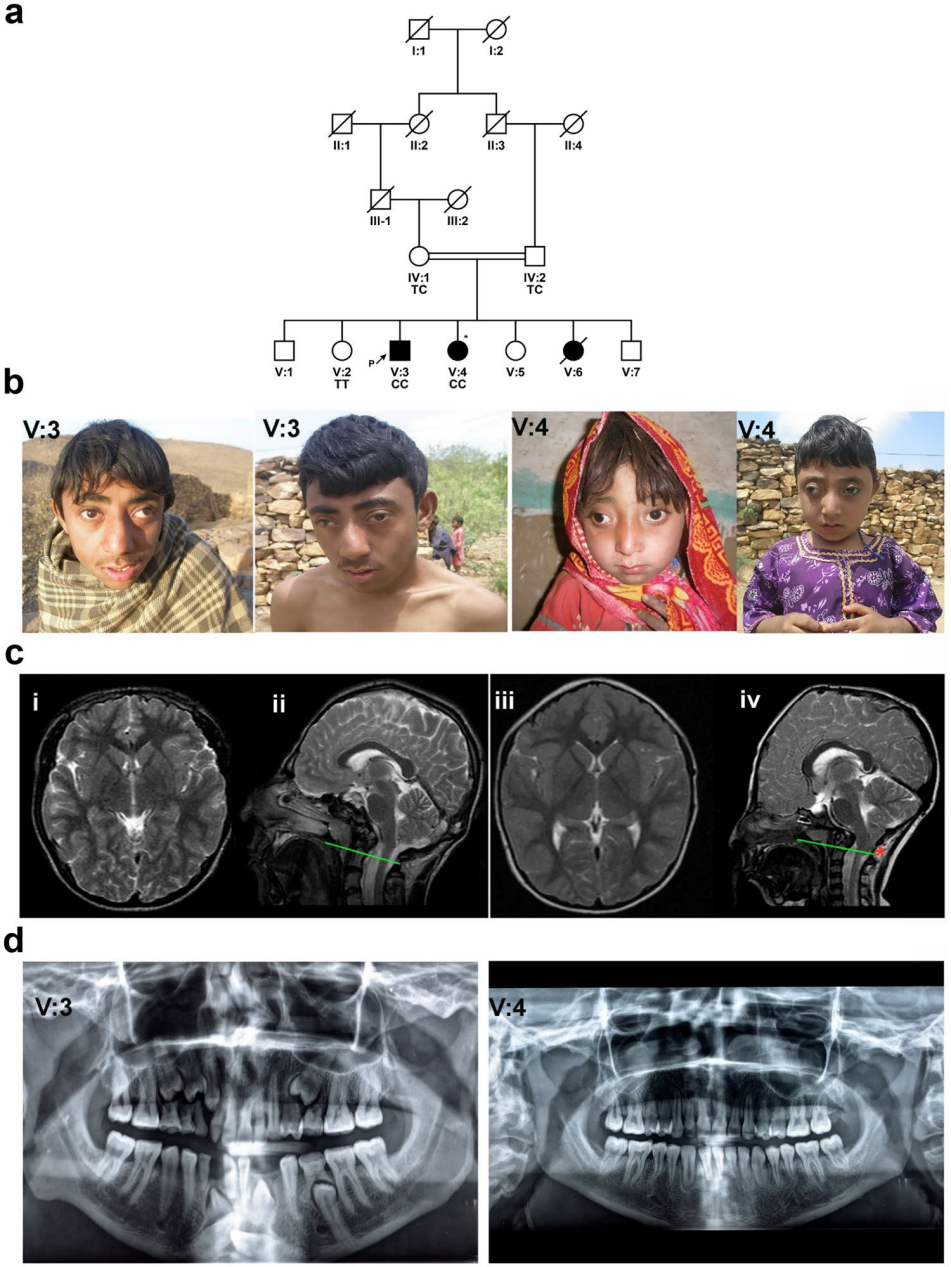
A craniosynostosis phenotype in a Pakistani family. (**a**) An extended consanguineous pedigree with three affected individuals who harbor the c.919 T > C variant. Filled black circles and squares, affected females and males, respectively; unfilled shapes, unaffected; crossed by a line represents a deceased individual. An index diagnosis is indicated by an arrowhead. An asterisk designates the individual on whom WES was performed. Genotypes are indicated below the genetic symbols of each examined member: TT (wild type), TC (heterozygous), and CC (homozygous mutant). (**b**) Facial views of live affected individuals of the Pakistani CS family (subjects V:3 and V:4 at age 13 and 9 respectively) (**c**). **i** and **ii**: axial and sagittal T2-weighted images of individual V:3 show brachycephaly, mild platybasia and a basilar invagination. **iii** and **iv**: axial and sagittal T2 weighted images of individual V:4 reveal trigonocephaly and increased transverse diameter, * indicates Chiari-like malformation with cerebellar tonsils extending below the foramen magnum, platybasia and a degree of basilar invagination. (**d**) Orthopantomograph of the individual (V:3) show over-retained deciduous teeth and erupting succedaneous permanent teeth. Individual (V:4) had no anomaly on the teeth.

The three affected individuals in the pedigree were born to healthy first-cousin parents (Fig. 1a), consistent with autosomal recessive inheritance. At birth, all three were found to have an unusual head shape. Individual V:6 died in early childhood with no documented history. At the age of 13 years, the proband (V:3) was suspected to have craniosynostosis due to the abnormal head shape by the local physician (Fig. 1b). The parents were asked to have an MRI (magnetic resonance imaging) of the brain on both children for further evaluation of craniosynostosis. The MRI scan of the individual (V:3) revealed brachycephaly with shortened anteroposterior (OFC) skull length as well as platybasia and basilar intussusception (Fig. 1c). His permanent teeth had all developed with retained deciduous dentition (Fig. 1d). He had a flattened head, exophthalmos, protruding chest, a broad nose, and a reduced head circumference. In addition, he had significant visual impairment (count fingers vision). His sister (V:4) was diagnosed at 9 years of age and had the same phenotypic spectrum (Fig. 1b–d). She had a fused metopic suture, reduced occipitofrontal circumference (OFC), and was trigonocephalus. In addition, she had a short Chiari-like malformation, a bossing forehead, beaked nose, and short philtrum with a protruding rib cage. Hand, ear, hearing, and feet were normal in both affected indviduals. As a limitation, it has to be noted that the diagnosis of craniosynostosis was made by clinical exam rather than a CT scan. The physical and mental development of both individuals was appropriate for their age. All clinical details are summarized in Table 1.

**Table 1:** Clinical details of the family with CS.

Subject ID	Sex	Age (years)	age at diagnosis (years)	Intellectual disability	Synostosis type	Skull shape	Delayed Tooth eruption	Forehead	Height (at age)	HC	Hearing impairment	Vision impairment	Limb malformation	Other assocaited phenotypes	Health status	*IL11RA* Genotype
IV:1	M	64	–	No	–	–	–	–	–	–	No	reduced vision	No	–	U	TC
IV:2	F	55	–	No	–	–	–	–	–	–	No	No	No	–	U	TC
V:1	M	31	–	No	–	–	–		–	–	No	No	No	–	U	-
V:2	F	27	–	No	–	–	–		–	–	No	No	No	–	U	TT
V:3	M	23	13	No	Coronal synostosis	Brachycephaly	yes	Protruding	159 cm (13 y)	48 cm (13 y)	No	CF	No	uneven rib cage	A	CC
V:4	F	19	9	No	Metopic synostosis	Trigonocephalus	yes	Protruding	101 cm (9y)	44 cm (9y)	No	impaired vision	No	protruding rib cage	U	CC
V:5	F	15	–	No	–	–	–	–	–	–	No	No	No	No	U	–
V:6	F	12	–	No	–	–	–	–	–	–	No	No	No	No	A	–
V:7	M	9	–	No		–	–	–	–	–	No	No	No	No	U	–

### Identification of a homozygous variant in IL11RA

**Figure 2:**
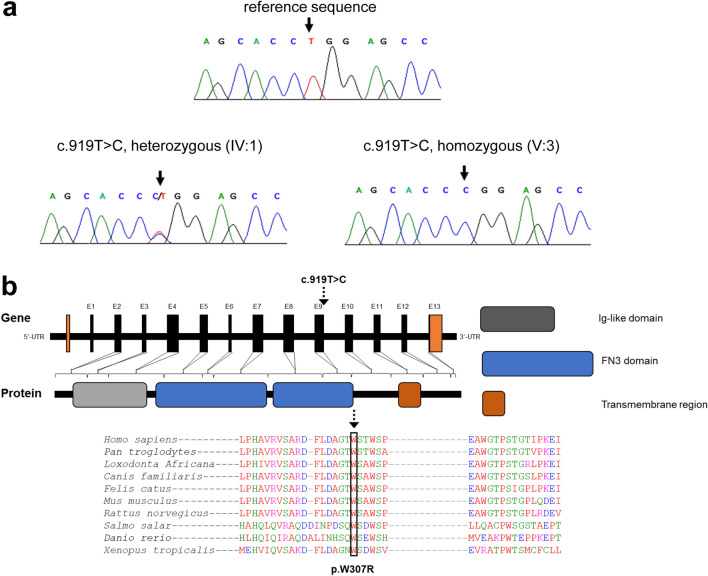
A homozygous missense c.919 T > C/p.W307R variant in *IL11RA* is present in members of the family. (**a**) Representative sequence electropherograms of wild type sequence and of c.919 T > C in heterozygous (IV:1) and homozygous (V:3) individuals. (**b**) Schematic of the human *IL11RA* gene and protein domain structure. The IL-11R protein comprises an immunoglobulin (Ig)-like, two fibronectin type 3 domains (FN3)-like (D2 and D3), a flexible stalk region, a transmembrane (TM) helix and a short intracellular part. Position of (c.919 T > C, p.W307R) is indicated. Conservation of the residue is determined by multiple sequence alignments of *IL11RA* across different species using Clustal Omega^62^.

To determine the underlying genetic cause of the phenotype, whole-exome sequencing (WES) was performed on the individual V:4. Based on the consanguinity of the healthy parents, an autosomal recessive mode of inheritance was inferred. After applying the previously described filtering criteria^43,44^, a homozygous missense variant was identified in exon 9 of the interleukin-11 receptor subunit alpha (*IL11RA*; NM_001142784.2) gene at position c.919 T > C (Fig. 2a and supplementary file S1). This variant results in the substitution of the tryptophan at position 307 by an arginine, p.W307R. Tryptophan 307 is highly conserved in IL-11R orthologues and is located in the second fibronectin type III (FNIII) domain of the IL-11R (Fig. 2b). No other putative disease-causing alleles were identified in genes previously associated with craniosynostosis. This variant was absent from all publicly available exome/genome databases and from the CCG in-house dataset (> 3360 exomes). Evaluation of this amino acid position using variant pathogenicity prediction tools (CADD_phredscore, 26.9^51^; dbNSFP_score, 0.8^52^; SIFT_score, 0.0^53^; PolyPhen_score, 0.9^54^; LRT_score, 0.0; and VEST3_score, 0.92^55^) suggests that the observed change is protein damaging. Sanger sequencing of all available DNA samples from the family members revealed co-segregation of the variant with the phenotype according to the supposed recessive genetic model (Fig. 1a). According to the 2015ACMG/AMP guidelines, this variant was rated to be pathogenic (PM1, PM2, PP1, PP3, PS3) and disease-causing.

### The p.W307R variant is located in domain D3 of the IL-11R and prevents IL-11R maturation

**Figure 3:**
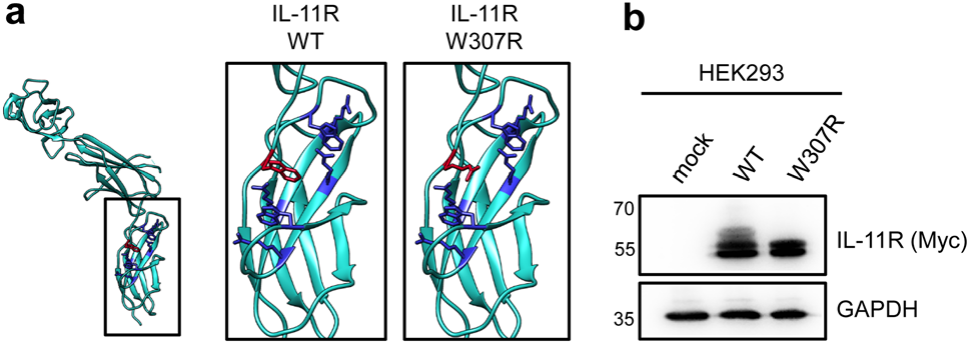
The p.W307R variant is located in the arginine-tryptophan-zipper within the domain D3 of the IL-11R and prevents IL-11R maturation. (**a**) Structure of the extracellular part (domains D1, D2 and D3) of the IL-11R according to the published crystal structure^50^ (pdb accession code 6O4P). Within the D3 domain (enclosed), the amino-acid residues which constitute the arginine-tryptophan-zipper are highlighted in dark blue with the exception of W307, which is shown in dark red. Magnification of the D3 domain is shown in the insets on the right hand site of the total structure, which represent the WT situation (W307, left inset) and the patient variant (R307, right inset). (**b**) HEK293 cells were transiently transfected with expression plasmids encoding IL-11R WT, IL-11R-W307R or mock control. 48 h later, cells were lysed and expression of the IL-11R variants was analyzed by western blot. GAPDH was visualized on the same membrane to verify the loading of equal protein amounts. One representative experiment out of three experiments with similar outcome is shown.

The IL-11R consists of three extracellular domains, of which the D2 and D3 domains are required for binding of the ligand IL-11. Pathogenic variants linked to craniosynostosis are described in all three domains^18^. We have previously characterized the missense patient variant R296W of the IL-11R and shown that R296 is part of the arginine-tryptophan-zipper, an important structural element of alternating arginine and tryptophan residues crucial for proper folding of the D3 domain^41^. Using the recently published structure of the extracellular part of the IL-11R^50^, we realized that W307 is part of the same arginine-tryptophan-zipper (Fig. 3a). We therefore reasoned that the W307R variant found in the patients could potentially also lead to misfolding or destabilization of the IL-11R in a similar manner, which would also explain the results obtained with the mutation pathogenicity prediction tools. In order to investigate this further, we introduced the variant into an expression plasmid encoding an N-terminally myc-tagged IL-11R that we have used previously to analyze IL-11R biology^41,42,48^. We transiently expressed both IL-11R-WT and IL-11R-W307R in HEK293 cells and analyzed IL-11R expression via western blot. As shown in Fig. 3b, IL-11R-WT was detected in at least three bands of different molecular weight, which is in line with our previous observation that these bands originate from differential glycosylation of the receptor^56^. Importantly, the upper band with the highest molecular weight corresponds to the fully matured IL-11R at the cell surface^41,56^. Intriguingly, this upper band was completely absent in HEK293 cells transfected with IL-11R-W307R, indicating that maturation of this IL-11R variant is impaired (Fig. 3b). These results confirm the importance of the intact arginine-tryptophan-zipper for maturation of the IL-11R and suggest that the p.W307R variant disrupts the zipper, thereby compromising the folding of the IL-11R.

### IL-11R-W307R is not transported to the cell surface, but is retained intracellularly

**Figure 4:**
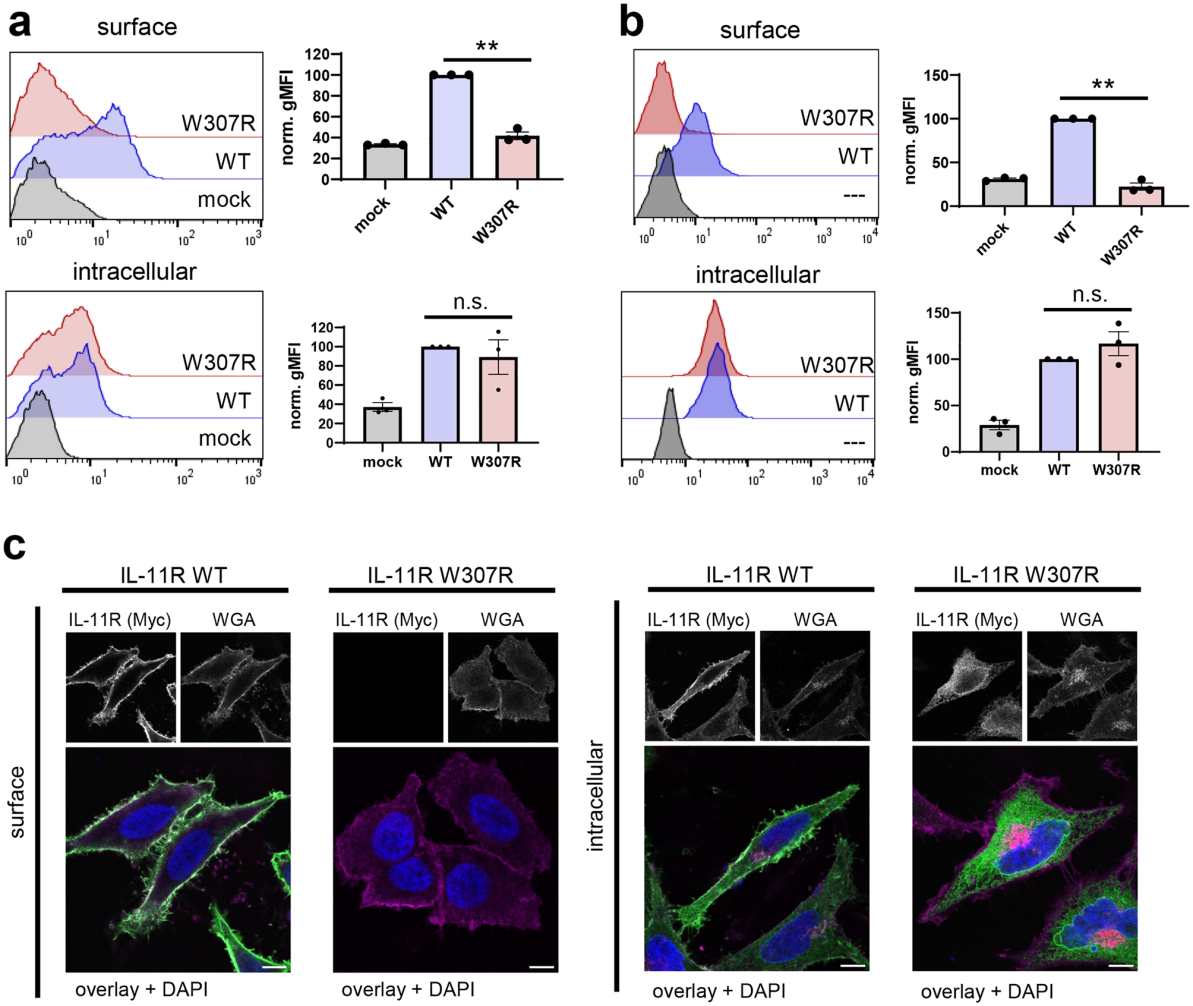
IL-11R-W307R is not transported to the cell surface, but retained intracellularly. (**a**) HEK293 cells were transiently transfected with expression plasmids encoding IL-11R WT, IL-11R-W307R or mock control. 48 h later, cells were harvested and the amount of IL-11R was assessed via flow cytometry. Prior to the antibody staining, cells were either left untreated for cell surface staining (“surface”, upper panel) or permeabilized using saponin for intracellular staining (“intracellular”, lower panel). The geometric mean fluorescence intensity (gMFI) of HEK293 cells expressing IL-11R-WT was set to 100, and the gMFI values of the other cells were calculated accordingly. One experiment out of three is shown on the left side, while quantification of three independent experiments (mean ± SEM) is shown on the right side. Statistical analysis was performed using one sample T test between IL-11R WT and IL-11R-W307R-expressing cells: **:* p* < 0.01; n.s.: not significant. (**b**) The experiment was performed with Ba/F3-gp130, Ba/F3-gp130-IL-11R-WT and Ba/F3-gp130-IL-11R-W307R as described for panel a. The geometric mean fluorescence intensity (gMFI) of Ba/F3-gp130-IL-11R-WT cells was set to 100, and the gMFI values of the other cells were calculated accordingly. One experiment out of three is shown on the left side, while quantification of three independent experiments (mean ± SEM) is shown on the right side. Statistical analysis was performed using one sample T test between IL-11R-WT and IL-11R-W307R-expressing cells: **:* p* < 0.01; n.s.: not significant. (**c**) HeLa cells were transiently transfected with expression plasmids encoding either IL-11R WT or IL-11R-W307R. IL-11R was stained with an anti-myc antibody, and cells were either permeabilized or not before IL-11R staining to visualize total IL-11R expression or just the IL-11R at the cell surface. Cellular membranes were visualized with WGA, cell nuclei with DAPI. Staining of IL-11R/myc is shown in green, WGA in purple and DAPI in blue. Representative images from three independent experiments with similar outcome are shown. Scale bar: 10 µm.

In order to further analyze the consequences of the incomplete IL-11R maturation, we determined the amount of IL-11R-WT and IL-11R-W307R at the cell surface via flow cytometry. Our previous study had shown that IL-11R-R296W was not transported to the cell surface, but rather retained intracellularly^41^, and we were therefore interested to see whether the p.W307R variant would cause a similar effect. Indeed, while IL-11R-WT was clearly detectable at the cell surface of transiently transfected HEK293 cells, the amount of IL-11R-W307R was significantly lower and indistinguishable from mock-transfected control cells (Fig. 4a). Importantly, when we permeabilized the cells and stained IL-11R intracellularly, we could clearly detect both IL-11R-WT and IL-11R-W307R at similar levels, underlining that the p.W307R variant did not simply prevent expression of the IL-11R, but obviously hinders its transport to the cell surface (Fig. 4a). In order to not only rely on transient overexpression, we stably transduced Ba/F3-gp130 cells with either IL-11R-WT or IL-11R-W307R and determined also here the amount of IL-11R at the cell surface via flow cytometry. In line with our results with the HEK293 cells, we could detect high amounts of IL-11R at the cell surface of Ba/F3-gp130-IL-11R-WT cells (Fig. 4b). The amount of IL-11R on Ba/F3-gp130-IL-11R-W307R cells did not differ from the untransduced parental Ba/F3-gp130 cells and was of course significantly reduced compared to Ba/F3-gp130-IL-11R-WT cells, indicating that also in this cell line IL-11R-W307R was unable to reach the cell surface. Intracellular staining of IL-11R in both cell lines again showed that both IL-11R variants were expressed at similar amounts, further substantiating the notion that the p.W307R variant influences intracellular transport, but not expression of the IL-11R (Fig. 4b).

Finally, we also expressed both proteins transiently in HeLa cells, stained IL-11R at the cell surface and visualized them by confocal microscopy. While IL-11R-WT was clearly detectable at the cell surface, we did not detect IL-11R-W307R, confirming the observation that maturation of IL-11R-W307R and subsequently also the transport to the cell surface was impaired (Fig. 4c). Like in the other experiments, permeabilisation and thus the visualization of intracellular IL-11R showed equal expression of both IL-11R-WT and IL-11R-W307R (Fig. 4c). We concluded from these experiments that W307R not only prevents maturation of the IL-11R, but also hinders the transport of the IL-11R to the cell surface.

### Cells expressing IL-11R-W307R do not respond to IL-11 stimulation

**Figure 5:**
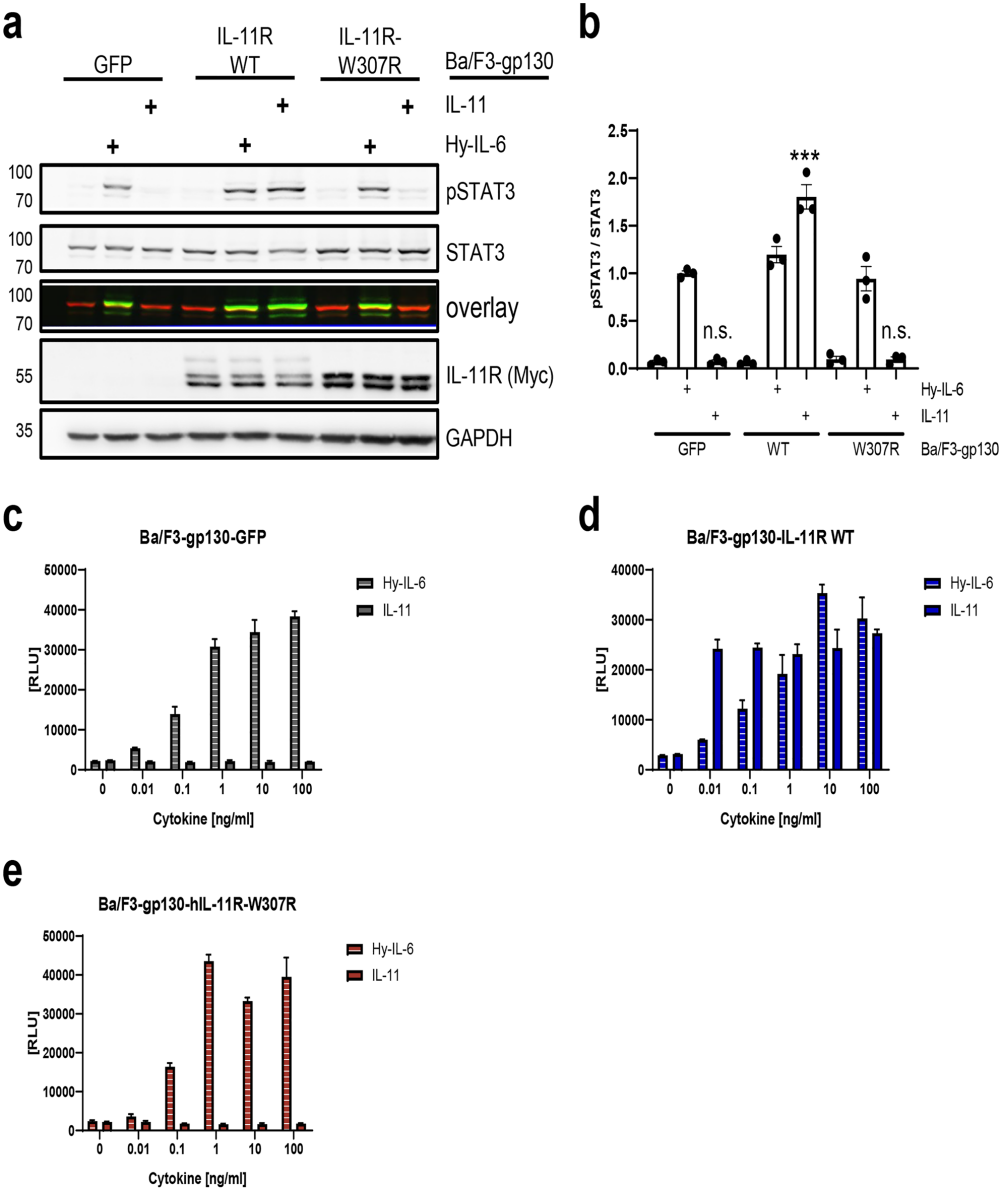
Cells expressing IL-11R-W307R do not respond to IL-11 stimulation. (**a**) Equal amounts of Ba/F3-gp130-GFP, Ba/F3-gp130-IL-11R-WT and Ba/F3-gp130-IL-11R-W307R cells were serum-starved for 120 min and then stimulated for 15 min with either 10 ng/ml IL-11, 10 ng/ml Hyper-IL-6 (Hy-IL-6) or left unstimulated. Cells were directly boiled in Laemmli buffer afterwards and pSTAT3, total STAT3, IL-11R and GAPDH were visualized via western blot. One experiment out of three with similar outcome is shown. (**b**) Quantification of the three experiments described in panel a (mean ± SEM). Shown is the signal intensity of phosphorylated STAT3 in relation to to the signal intensity of total STAT3. (**c**–**e**) Equal amounts of (**c**) Ba/F3-gp130-GFP, (**d**) Ba/F3-gp130-IL-11R-WT and (e) Ba/F3-gp130-IL-11R-W307R cells were incubated with increasing amounts of either Hyper-IL-6 (HY) or IL-11 (0.01–100 ng/ml) or left untreated. 48 h later, cell viability was determined as described in Materials and Methods. Shown is one experiment (n = 3 technical replicates, mean ± SEM) out of three experiments performed with similar outcome.

Finally, we analyzed the functional consequences of the impaired maturation of the IL-11R-W307R variant. For this, we used the generated Ba/F3-gp130 cell lines. Ba/F3 cells are murine pre-B cells that are only viable in the presence of the cytokine IL-3 and undergo apoptosis without an appropriate stimulus. Ba/F3-gp130-IL-11R cells stably expressing gp130 and IL-11R do not need IL-3 for proliferation, but can be stimulated with IL-11 instead, making this cell line an ideal tool to study mutations in cytokine receptors. Therefore, we treated Ba/F3-gp130-GFP, Ba/F3-gp130-IL-11R-WT and Ba/F3-gp130-IL-11R-W307R cells with either 10 ng/ml IL-11 or 10 ng/ml Hyper-IL-6 for 15 min or left them unstimulated and determined phosphorylation of the transcription factor STAT3, which is downstream of gp130, via western blot. Hyper-IL-6 is a fusion protein of IL-6 and the soluble IL-6R that can activate gp130 directly without the need for a functional alpha receptor and can therefore be used as a positive control for all three cell lines^46^. As expected, we observed STAT3 phosphorylation in Ba/F3-gp130-GFP cells only when they were treated with Hyper-IL-6, but neither in untreated nor IL-11-stimulated cells (Fig. 5a,b). Further, STAT3 phosphorylation in Ba/F3-gp130-IL-11R-WT cells was induced by both Hyper-IL-6 and IL-11, indicating that both receptors were biologically active (Fig. 5a,b). Importantly, Ba/F3-gp130-IL-11R-W307R responded to Hyper-IL-6, but not to IL-11 stimulation, confirming that the impaired cell surface amount of the IL-11R renders this cell line unresponsive towards IL-11 stimulation (Fig. 5a,b). Furthermore, the western blot of cell lysates of the three cell lines showed three distinct IL-11R bands for Ba/F3-gp130-IL-11R-WT, but only two bands for Ba/F3-gp130-IL-11R-W307R, further confirming the impaired maturation we had observed in the HEK293 cells (Figs. 3b, 5a). Intriguingly, the two bands for the IL-11R-W307R had a higher intensity compared to the corresponding bands of IL-11R-WT, underlining impaired maturation and thus intracellular accumulation of IL-11R-W307R (Fig. 5a).

Having shown that downstream signaling is not functional in the Ba/F3-gp130-IL-11R-W307R cells, we also assessed long-term consequences of the variant. Cell viability can be used as a direct surrogate for cell proliferation in Ba/F3 cells, and we therefore incubated all three Ba/F3-gp130 cell lines with increasing amounts of either IL-11 or Hyper-IL-6 (0–100 ng/ml) for 48 h and determined cell viability afterwards. Ba/F3-gp130-GFP cells proliferated when stimulated with Hyper-IL-6 in a dose-dependent manner, while stimulation with IL-11 did not induce any proliferation (Fig. 5c). As expected, Ba/F3-gp130-IL-11R-WT cells proliferated dose-dependently when stimulated with either Hyper-IL-6 or IL-11 (Fig. 5d). In line with our previous results, Ba/F3-gp130-IL-11R-R307W cells did not respond to IL-11 stimulation at all, but proliferated with Hyper-IL-6 stimulation (Fig. 5e). In summary, these results show that the p.R307W variant renders cells unresponsive to IL-11, even at very high amounts of up to 100 ng/ml IL-11, which can be explained by lack of transport of the IL-11R to the cell surface.

## Discussion

Premature closure and ossification during skull development of one or several of the sutures, flexible structures that line the calvaria and ensure proper expansion of the skull, is termed craniosynostosis. Depending on the severity of the phenotype, affected children have to undergo surgery to re-open the sutures, and craniosynostosis can be associated with blindness, deafness and mental retardation, e.g. caused by increased intracranial pressure due to the inability to cope with the expanding brain^2^. The complex process of skull development requires the coordination of multiple cell types and signaling pathways in a timely and spatial manner. It is therefore logically consistent that variants in proteins involved in these signaling pathways, which result in an alteration of the signaling output, disrupt this highly organized developmental process and result in a clinical phenotype. Understanding how these variants affect the signaling pathways is therefore essential for developing therapeutic strategies to help the affected patients.

Until today, more than 30 patients have been described that present with craniosynostosis and have a variation within the IL-11R^18^. The majority of these variants are missense variants, in which one amino acid residue is exchanged by another, but also small deletions, insertions and premature stop codons have been reported^18^. All known variants are located within the extracellular part of the receptor, which underlines that the the transmembrane helix and the intracellular part are not strictly required for IL-11 signaling, as the initiation of intracellular signaling cascades is executed by the β-receptor gp130. Furthermore, signaling via soluble forms of the IL-11R (sIL-11R) constitutes IL-11 trans-signaling^48,57,58^, and sIL-11R does not contain the transmembrane domain and the intracellular region. However, as long as such mutations have not been identified in humans, it remains possible that alterations within the transmembrane domain and the intracellular region can cause a phenotype. A significant number of variants are specifically located within the D3 domain^18^, which is reported to be the domain that binds IL-11 with high affinity^59^. One would expect that variants in the ligand-binding part have a significant functional impact.

The novel pathogenic variant p.W307R described in this manuscript has to our knowledge not been reported previously in the literature. Our results clearly show that the IL-11R-W307R variant is expressed at amounts comparable to IL-11R-WT, but that it does not mature correctly, is retained within the cell, most likely within the endoplasmic reticulum, and does not reach the cell surface. Because the ligand IL-11 is located in the extracellular space and binds to the IL-11R at the cell surface, the absence of IL-11R at the plasma membrane results in a complete loss of IL-11 signaling. We have previously described that a structural element within the D3 domain of the IL-11R, called an arginine-tryptophan-zipper, is crucial for stability and folding of the IL-11R and that disruption of this zipper, which was exemplified by the p.R296W variant, results in intracellular retention of the IL-11R^41^. Intriguingly, W307 is also part of this zipper motif, and we can therefore conclude that the p.W307R variant causes a similar folding problem of the D3 domain and is the molecular reason for the observed maturation defect for IL-11R-W307R.

Interestingly, until now, only craniosynostosis patients with variants within the *IL11RA* gene have been described, but no patients with variants in *IL11*, which encodes the cytokine itself, are known. This is rather surprising, given the fact that a non-functional IL-11 should result in a similar human phenotype. However, until now, only a coding single nucleotide polymorphism within *IL11* is known, which results in decreased stability of IL-11^47^ and is associated with reduced human height in adults^60^. The reason for the lack of craniosynostosis in such patients is currently unclear. Either these patients have not been clinically seen yet, or a lack of IL-11 in vivo can be compensated by a second cytokine that can signal through the IL-11R. A recent study did not find craniosynostosis in an IL-11 knock-out mouse strain, which would argue for the second hypothesis^61^, but the identity of such a cytokine is currently unknown.

In conclusion, we describe a novel craniosynostosis-associated pathogenic variant within the IL-11R and elucidate the molecular mechanism underlying the dysfunction of this IL-11R variant. Future work should focus on the question of how the lacking IL-11 signaling in such a patient can be restored. This could either be achieved by forcing the mutated receptor out of the ER and to the cell surface, as long as the receptor is in principle functional and only mislocated within the cell. Other approaches could include designer cytokines that stimulate the required signaling pathways and do not require the presence of a functional membrane-bound IL-11R.

### Supplementary Information

Supplementary Information 1.

Supplementary Information 2.

## Data Availability

The variant described in this paper has been deposited in the ClinVar database (accession number: SCV003798490.1; https://www.ncbi.nlm.nih.gov/clinvar/variation/2429382/?oq=SCV003798490). All other data generated or analysed during this study are included in this published article (and its Supplementary Information files).
