# Supplementary material for: The interleukin-11 receptor variant p.W307R results in craniosynostosis in humans
Source: Sci Rep. 2023 Aug 18;13:13479. doi: 10.1038/s41598-023-39466-y (PMC10439179; doi:10.1038/s41598-023-39466-y)
Supplement: Supplementary file 1 — Supplementary Information 1. [file 41598_2023_39466_MOESM1_ESM.pdf]

Supplementary Material S1

1. Whole Exome Sequencing

Whole Exome Sequencing (WES) in CS patient: Variant filtering was performed using the latest version of VARBANK graphical user interface (https://varbank.ccg.uni-koeln.de/varbank2, accessed on 10 January 2023). Employing the standard filter criteria for rare and homozygous variants: Coverage > 5 reads; Quality Score > 10; Allele Read Frequency ≥ 75%; Minor Allele Frequency (MAF) < 0.001 in gnomAD; in-house population allele frequency < 0.001; Strand Bias Estimated using Fisher's Exact Test (FS) < 40; MQRankSum ≥ −5; QD > 5; MQ > 50; ReadPosRankSum ≥ −5; quorum level indel = 1; mapping quality > 50; quorum level SNV = 2; splice site score change ≤ 15%; and translation initiation site (+score change > 15%, −score change −15%), 202 different genetic variants (97 SNVs,73 CNVs, and 32 indels) were filtered in the first step. The resulting gene lists were prioritized based on scores obtained from the dbNSFP/dbSCSNV v3.4 databases (filtering was based on normalized rank scores ranging from 0 = benign to 1 = pathogenic) and variants located in regions of runs of homozygosity [ROH, (~3 Mb]. To further narrow the search for disease-causing variants, coding variants (single-nucleotide variants (SNVs) and insertions or deletions (InDels) that could have a damaging effect on protein structure or function, as well as canonical splice site variants (i.e., splice site donor and splice site acceptor variants) were retained and the rest were discarded. In the final step, the disease-causing variant was selected based on the OMIM database; dbNSFP score >0.5, CADD Phred score >20, Polyphen2 score >0.6, SIFT scores <0.5, and literature research. In addition, data from several public databases (dbSNP154, 1000 Genomes, and Greater Middle Eastern Variome) were used to determine the distribution of genetic variants in large populations, and disease-specific databases (commercial HGMD professional database and ClinVar) were searched to determine whether variants were associated with phenotypes.

(a) (b)

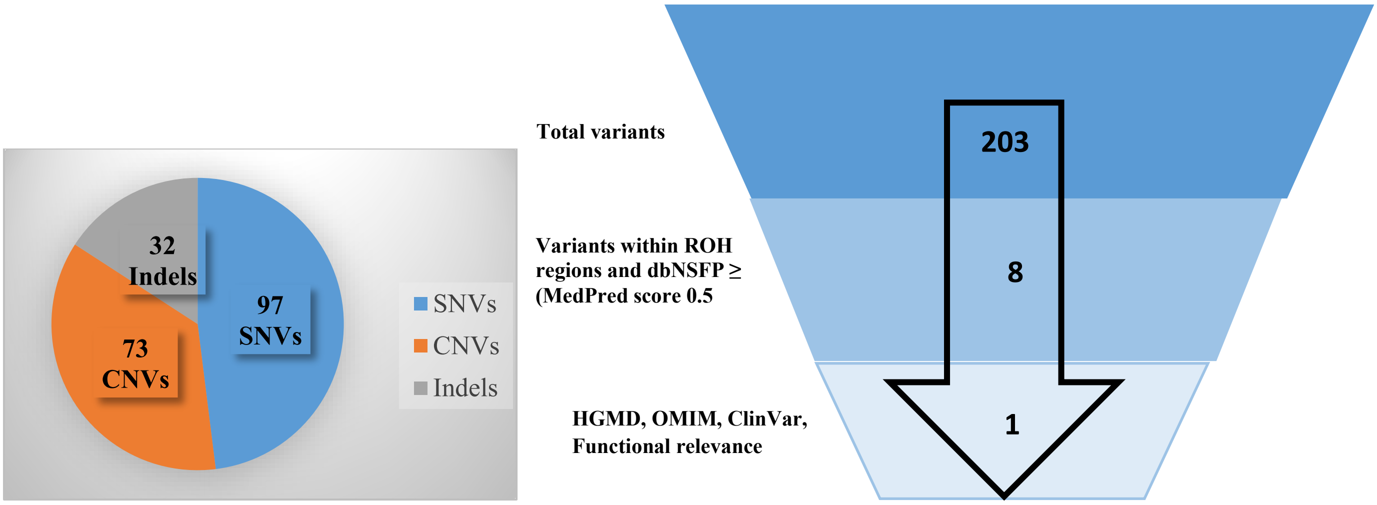

Variant filtering using Varbank

Supplementary Figure 1. (a) Overview of the different rare homozygous variants after assuming autosomal recessive monogenic inheritance using web interface of Varbank 2. (b) Filtering steps to find disease causing variant.

Supplementary table 1: List of homozygous variants.

Chr:region (build GRCh38/hg38)	Gene	Mut cDNA (Mut Prot)	MedPred	gnomAD Frequency	dbSNP annotation	CADD PHRED V1.3	Poly-phene 2	SIFT	disease association (OMIM number, mode of inheritance)
1: 46859589	*CRYZ*	c.589G>A (p.Val197Met)	0.64	0.00001993	rs766868441	27.3	0.89	0.06	NA, but a susceptibility gene for amyotrophic lateral sclerosis
7:116700039	*TRRAP*	c.6461T>C (p.Met2154Thr))	0.64	0.0000650	rs772549634	14.46	0.01	0.05	Developmental delay with or without dysmorphic facies and autism, Deafness (MIM: 618778, 618454; AD)
8:144505342	*GPT*	c.592G>C (p.Glu198Gln)	0.67	0.0005430	rs530505425	22.6	0.98	0.65	NA
12:95108510	*FGD6*	c.3185A>G (p.Lys1062Arg)	0.71	0.001058	rs143209934	22.6	0.53	0.23	NA
13:23341379	*SACS*	c.2497G>A (p.Glu833Lys)	0.72	0.0001446	rs143433500	24.2	0.94	0.36	NA
**chr9:34659867**	***IL11RA***	**c.919T>C (p.Trp307Arg)**	**0.80**	**-**	**-**	**26.9**	**1.0**	**0.0**	**Craniosynostosis and dental anomalies (MIM: 614188, AR)**
3:151445827	*IGSF10*	c.4154C>G (p.Ser1385*)	-	0.0001074	rs770578800	-	-	-	NA
X:48347610	*SSX3*	c.467-6T>A	-	0.003691	rs782716495	-	-	-	NA

Supplementary table 1: List of homozygous candidate variants in ROH regions. These variants are located within regions of homozygosity which are derived from exome sequence data. The CADD_phred, SIFT, PolyPhen, and MedPre scores show the in-silico pathogenicity prediction of the variant. Variants highlighted in bold represent the causative variant for the phenotype under investigation. NA = not available, NV= single nucleotide variant, AR= autosomal recessive, AD= autosomal dominant. These variants are segregating with the phenotype in the family. *IL11RA* is located within the ROH region (GRCh38chr9:27,567,147-74,738,384, size 47.1 MB).

2. Score of each algorithm used by dbNSFP to compile the prediction score

SIFT_score: 0.0; Polyphen2_HDIV_score, 1; LRT_score, 0; MutationTaster_score, 0.99; MutationAssessor_score, 2.89; FATHMM_score, -1.2; PROVEAN_score, -12.45; VEST3_score, 0.92; MetaSVM_score, 0.53; Reliability_index, 10; M-CAP_score, 0.23; REVEL_score, 0.84; MutPred_score, 0.84; CADD_phred, 26.9; DANN_score, 0.99; fathmm-

MKL_coding_score, 0.93; Eigen-phred, 8.13; GenoCanyon_score, 0.99; integrated_fitCons_score, 0.65; GM12878_fitCons_score, 0.61; H1-hESC_fitCons_score, 0.67; HUVEC_fitCons_score, 0.65; GERP++_NR, 5.48; GERP++_RS_rankscore, 0.8; phyloP100way_vertebrate, 5.28; phastCons100way_vertebrate, 0.71; SiPhy_29way_pi, 0.0; SiPhy_29way_logOdds_rankscore, 0.60.

Online Web Resources

gnomAD (https://gnomad.broadinstitute.org)

1000 genome (http://browser.1000genomes.org)

dbSNP155 (https://ftp.ncbi.nlm.nih.gov/snp/)

Greater Middle Eastern Variome (http://igm.ucsd.edu/gme/index.php)

CADD Phred score (http://cadd.gs.washington.edu/)

Variant Effect Predictor (https://www.ensembl.org/Tools/VEP)

Clustal Omega (https://www.ebi.ac.uk/Tools/msa/clustalo/)

VARBANK pipeline (https://varbank.ccg.uni-koeln.de)

Online Mendelian Inheritance in Man (OMIM) (www.omim.org)

ClinVar, (http://www.ncbi.nlm.nih.gov/clinvar)

Human Gene Mutation Database, (http://www.hgmd.org)
