# Supplementary figures and images for: The interleukin-11 receptor variant p.W307R results in craniosynostosis in humans
Source: Sci Rep. 2023 Aug 18;13:13479. doi: 10.1038/s41598-023-39466-y (PMC10439179; doi:10.1038/s41598-023-39466-y)

Figure 3b

Myc (IL-11R)

GAPDH

Myc + GAPDH

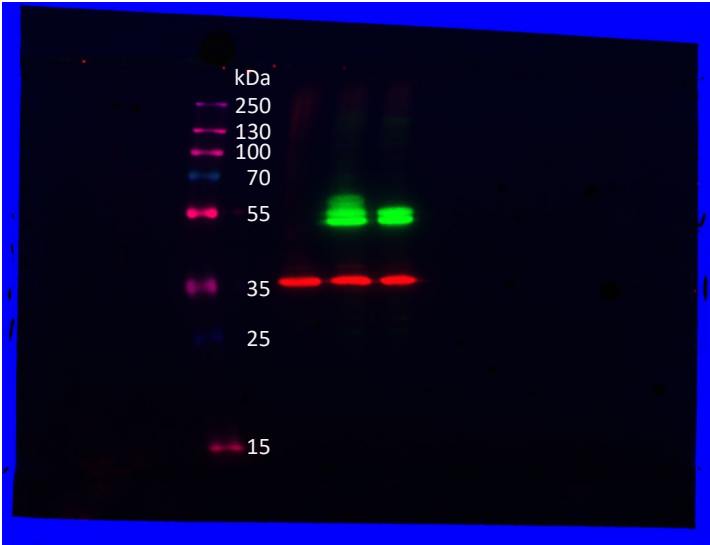

Figure 5a

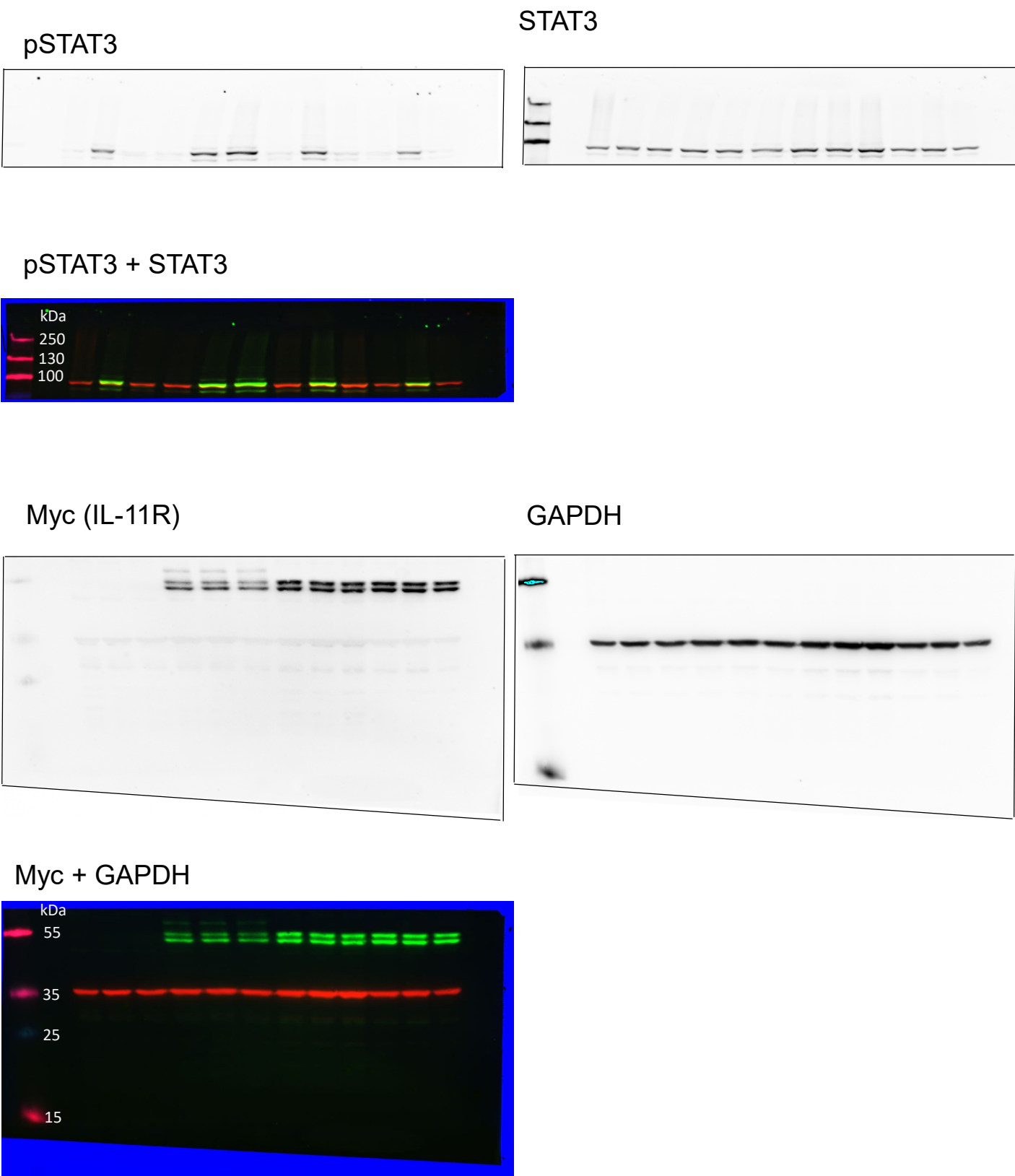

Supplement: Supplementary file 2 — Supplementary Information 2. [file 41598_2023_39466_MOESM2_ESM.pdf]
